# The Contingency of Reported sST2 Serum Concentrations with a Protein Detection System (ELISA) from the Same Manufacturer (R&D Biotechne, 2002–2025): An Explanatory Effort by Applied Medical Researchers

**DOI:** 10.3390/diagnostics15182412

**Published:** 2025-09-22

**Authors:** Marie-Therese Lingitz, Hannes Kühtreiber, Lisa Auer, Michael Mildner, Bernhard Moser, Christine Bekos, Clemens Aigner, Martin Direder, Thomas Mueller, Hendrik Jan Ankersmit

**Affiliations:** 1 Division of General Anesthesia and Intensive Care Medicine, Department of Anesthesia, Critical Care and Pain Medicine, Medical University of Vienna, 1090 Vienna, Austria; 2 Department of Thoracic Surgery, Applied Immunology Laboratory, Medical University of Vienna, 1090 Vienna, Austria; 3 Comprehensive Center for Chest Diseases, Medical University of Vienna, 1090 Vienna, Austria; 4 Aposcience AG, 1200 Vienna, Austria; 5 Department of Dermatology, Medical University of Vienna, 1090 Vienna, Austria; 6 Department of Obstetrics and Gynecology, Medical University of Vienna, 1090 Vienna, Austria; 7 Division of Trauma-Surgery, Department of Orthopedics and Trauma-Surgery, Medical University of Vienna, 1090 Vienna, Austria; 8 Department of Laboratory Medicine, Hospital Voecklabruck, 4840 Voecklabruck, Austria

**Keywords:** sST2, ELISA standardization, biomarker variability

## Abstract

**Background/Objectives**: Soluble ST2 (sST2) has gained recognition as a clinically relevant biomarker across a spectrum of inflammatory, cardiovascular, and respiratory conditions. However, the lack of assay standardization raises concerns about result comparability across platforms and studies. **Methods**: This study systematically evaluated serum sST2 concentrations measured with two ELISA systems—DuoSet and Quantikine—produced by the same manufacturer (R&D Systems, Minneapolis, MN, USA). **Results**: Using archived serum samples from healthy volunteers and marathon runners, we identified marked discrepancies: serum sST2 concentrations using the DuoSet recombinant standard were on average 4.3-fold higher than those using Quantikine (median 308.3 [106.6–608.6] vs. 71.5 [41.8–115.6] ng/mL). On the pre-coated Quantikine plate, using the DuoSet recombinant standard increased calculated concentrations 4.3-fold compared with the native Quantikine standard (median 308.3 [106.6–608.6] vs. 71.5 [41.8–115.6] ng/mL). On the manually coated DuoSet plate, the DuoSet standard yielded higher medians than the Quantikine standard (8.0 [5.6–11.3] vs. 5.0 [3.7–7.4] ng/mL). Furthermore, between-lot variability within the same ELISA platform resulted in concentration shifts from 0.09 [0.07–0.10] ng/mL (2016) to 1.17 [0.81–3.23] ng/mL (2023) using the same sample. Previously published studies also exhibited wide inter-study variability among healthy cohorts. **Conclusions**: These findings emphasize that current ELISA systems for sST2 are not standardized and that cross-study comparisons should be interpreted with caution. Until universal standardization is implemented, sST2 should primarily be used for within-study comparisons. This variability may limit the reliability of longitudinal sST2 assessment even in clinical settings.

## 1. Introduction

The protein ST2 (also termed interleukin-1 receptor-like 1) is an interleukin-1 receptor family member that exists in two isoforms: a membrane-bound form (ST2L) and a soluble form (sST2) [1]. ST2L is expressed on the surface of various cells and acts as a receptor for interleukin-33 (IL-33), playing a role in inflammation and immune responses [1,2]. sST2 is a truncated form of ST2L that lacks the transmembrane and intracellular domains of ST2L and is released into the bloodstream. Although the major source of sST2 in healthy and diseased individuals is not fully established, previous findings by Mildner et al. [3] suggest that alveolar epithelial cells in the lung represent a primary source under inflammatory conditions. sST2 has emerged as a promising biomarker in various disease states, including cardiovascular diseases, respiratory diseases, inflammatory and autoimmune diseases, infectious diseases, and cancer [1,2]. Plasma concentrations of sST2 have been associated with disease severity, prognosis, and response to therapy in many of these conditions [1,2,3]. As a result, sST2 measurement has gained increasing attention as a potential tool for risk stratification, prognosis, and monitoring of various diseases [1,2,4].

The initial enzyme-linked immunosorbent assay (ELISA) for the measurement of sST2 in human serum/plasma was developed in 2000 [5]. Since that time, various additional assays have been designed to measure sST2 plasma concentrations, and several are commercially available [1]. However, concentrations of sST2 obtained with different assays are not equivalent [1,2]. It is therefore known that the existing sST2 assays are not standardized. It is questionable whether sST2 serum concentrations measured with two ELISAs from the same manufacturer are comparable or not. Our research group has also published several studies investigating sST2 concentrations in blood in various medical conditions, mainly using two commercially available assays from the same manufacturer, namely the DuoSet ELISA and the Quantikine ELISA (both R&D Systems, Minneapolis, MN, USA) [6,7,8,9,10]. Over the years, we observed a wide range in the magnitude of sST2 serum concentrations in our own studies. Therefore, the additional question arises whether commercially available ELISAs will show changes in concentration levels in comparable populations over time when using kits from different production lots.

Therefore, the aim of this study was to test the comparability of sST2 serum concentrations measured with two ELISAs from the same manufacturer and to evaluate whether the measured plasma concentrations of sST2 in certain samples differ significantly when using different lots of assay kits.

## 2. Materials and Methods

### 2.1. Study Design

We had two different study questions and retrospectively performed four different types of data evaluations in this work:

Study question (1): Are the sST2 serum concentrations measured with two ELISAs from the same manufacturer comparable or not? To answer this question, we performed evaluations (1a) and (1b) as detailed later.

Study question (2): Do the measured serum concentrations of sST2 in certain samples differ significantly when using different lots of assay kits? To answer this question, we performed evaluations (2a) and (2b) as detailed later.

sST2 measurements were made with the DuoSet ELISA and the Quantikine ELISA. For all standard comparisons (Evaluations 1a, 2a, and 2b), no cross-kit reagent use was performed. Each ELISA was conducted using its own dedicated reagents as instructed by the manufacturer. In Evaluation 1b, recombinant standards from one kit were deliberately applied to the other assay system to evaluate their effect on final concentration values; however, assay-specific diluents were still used in all cases.

This study is a secondary analysis of previously collected, fully anonymized data. The original study by Bekos et al. [7] was conducted in accordance with the Declaration of Helsinki and approved by the Institutional Review Board (IRB) and the Institutional Committee on Studies (ICS) of the Medical University of Vienna, Vienna, Austria (Approval Code: EK1034/2012). Details of the study design and ethical approvals are described in the previously published work [7]. Informed consent was obtained from all participants in the original study. No additional invasive procedures were performed on human participants for the present analysis.

### 2.2. sST2 Measurement Using the DuoSet ELISA (Research Use Only, RUO)

The DuoSet enzyme-linked immunosorbent assay was performed as follows: The capture antibody was diluted in phosphate-buffered saline and used to coat a 96-well microplate, followed by overnight incubation at 2–8 °C. After washing and blotting, wells were blocked with Reagent Diluent Concentrate 2 (DY995) diluted 1:10 in deionized water, incubated at room temperature, and washed again. A standard curve (0.0312–2.000 ng/mL) was prepared, and samples were diluted 1:2 in the appropriate buffer. Standards and samples were added in duplicate, incubated to allow analyte binding, and the plate was washed. The biotinylated detection antibody was added, incubated, washed, and followed by streptavidin–horseradish peroxidase (HRP), with incubation and washing repeated. Color was developed with 3,3′,5,5′-tetramethylbenzidine (TMB) and stopped to yield yellow. Absorbance was read at 450 nm with a 540 nm reference, and sST2 concentrations were obtained by interpolating mean background-corrected values on the standard curve and multiplying by the 2× sample dilution factor

The manufacturer does not provide any imprecision data for the R&D DuoSet ELISA (DY523B-05) in the package insert. We thus performed our own imprecision study at three different analyte concentrations applied in duplicates using the formula for coefficient of variation (*CV*%) calculation as described below for intra-assay precision:

$$CV\%=\left(\frac{standard\;deviation\;\left(SD\right)}{mean}\right)\;\times \;100$$

For inter-assay precision, we conducted the ELISA the same way as previously described with three other analyte concentrations applied in duplicates on two different microplates, and used the formula as described below for inter-assay precision:

$$CV\%=\left(\frac{standard\;deviations\;of\;different\;runs\;\left(SD\right)}{mean}\right)\;\times \;100$$

We found an intra-assay imprecision of <2% and an inter-assay imprecision of <16% as detailed in Table 1.

### 2.3. sST2 Measurement by Using the Quantikine ELISA (Research Use Only, RUO)

The R&D Quantikine ELISA was conducted following the manufacturer’s instructions. The wash buffer was diluted to the working dilution, and all reagents and samples were equilibrated to room temperature. A standard curve (0.0312–2.000 ng/mL) was prepared by serial dilution, and samples were diluted 1:20 as recommended. Standards and samples were added in duplicate to the pre-coated microplate and incubated to allow sST2 binding. Plates were washed thoroughly (final wash with inversion and blotting), the enzyme-linked detection conjugate was added and incubated, and washes were repeated. TMB substrate was then applied, color development was timed per protocol, and the reaction was stopped with the supplied stop solution. Absorbance was read at 450 nm with a 540 nm correction. Concentrations were determined by interpolating the mean, background-corrected duplicate values on the standard curve and multiplying by the 20× dilution factor; results were recorded for all samples. Imprecision was assessed for the DuoSet ELISA: intra-assay precision from three concentrations measured in duplicate on the same microplate; inter-assay precision from three additional concentrations measured in duplicate and repeated in a separate run. Coefficients of variation were calculated as described previously; intra-assay imprecision was <6% and inter-assay imprecision was <8% (see Table 1).

### 2.4. Evaluation (1a)

To answer the question of whether sST2 serum concentrations measured with two ELISAs from the same manufacturer are comparable in a first approach, we used serum aliquots from the study described by Bekos et al. [7]. Patient samples were aliquoted and stored at −80 °C in 2016. On 31 May 2023, the aliquots were thawed. Although no formal stability testing was performed on these specific samples, they were continuously stored at −80 °C and subjected to a maximum of two freeze–thaw cycles, conditions under which sST2 has previously been shown to remain stable (e.g., Mueller et al. [1]). The aliquots were allowed to thaw completely at room temperature before proceeding with the analysis. We measured the thawed samples on the day of thawing using both the DuoSet ELISA (lot number P317293) and the Quantikine ELISA (lot number P352309). The measured values of the marathoners and the half marathoners (obtained from samples drawn before the run, directly after crossing the finish line, and 2–7 days after the marathon) were compared descriptively as median with interquartile range. No statistical tests were performed.

### 2.5. Evaluation (1b)

To answer the question of whether sST2 serum concentrations measured with two ELISAs from the same manufacturer are comparable in a second approach, the Quantikine ELISA assay (lot number P352309) was conducted according to the manufacturer’s protocol, utilizing the pre-coated Quantikine plate and reagents. Alongside the standard Quantikine assay procedure, a recombinant standard from the DuoSet kit was included to generate an alternative standard curve. After completing the assay, the serum ST2 concentrations were calculated in two different ways. First, the concentrations were determined using the Quantikine standard curve, which was created with the recombinant standard provided in the Quantikine kit. Second, the concentrations were calculated using the DuoSet standard curve, which was generated with the recombinant standard from the DuoSet kit (lot number P317293). For each serum sample, the ST2 concentrations derived from these two standard curves were compared purely descriptively. No statistical comparisons were conducted. As serum samples, aliquots of 20 healthy controls from the study by Bekos et al. [7] were used. In this study, healthy volunteers were included as sedentary controls. They had no signs of cardiovascular disease, did not take medication regularly, and had a sedentary lifestyle without regular exercise.

Thereafter, the DuoSet assay was conducted following the manufacturer’s protocol, utilizing a non-pre-coated plate. In addition to the standard DuoSet assay procedure, a recombinant standard from the Quantikine kit was included to generate an alternative standard curve. After completing the assay, the serum ST2 concentrations were calculated in two different ways. First, the concentrations were determined using the DuoSet standard curve, created with the DuoSet recombinant standard. Second, the concentrations were calculated using the Quantikine standard curve, which was generated with the Quantikine recombinant standard. For each serum sample, the ST2 concentrations derived from these two standard curves were compared purely descriptively. No statistical comparisons were conducted. As serum samples, the aliquots of 20 healthy controls from the study by Bekos et al. [7] were used.

### 2.6. Evaluation (2a)

To preliminarily assess whether measured serum concentrations of sST2 vary significantly between different assay kit lots, we analyzed serum aliquots from the study by Bekos et al. [7], which had not undergone any freeze–thaw cycles prior to analysis in 2016. We retrieved the original values of this publication (DuoSet ELISA, order number 48852677, lot number not known), measured in 2016. We reached out to the manufacturer and reviewed our laboratory records in an effort to identify the lot number used. However, due to the considerable amount of time that has passed, we were unfortunately unable to retrieve this information. In addition, we used the concentrations we obtained from the thawed samples in 2023 (see experiment 1a, DuoSet ELISA, lot number P317293). We compiled the 2016 and 2023 data into a structured format for direct comparison. The distribution of the old and newly measured values was purely descriptive using box and whisker plots. No statistical tests were performed.

### 2.7. Evaluation (2b)

To answer the question of whether the measured serum concentrations of sST2 in certain samples differ significantly when using different lots of assay kits in a second approach, we explored our published data on the DuoSet ELISA or the Quantikine ELISA (each with different lots in a time course) and compared the respective mean values in healthy subjects. Previously published studies that used the DuoSet ELISA or the Quantikine ELISA were gathered. Studies reporting mean sST2 values for healthy controls were identified for inclusion. Studies were included if they reported mean sST2 concentrations specifically for healthy controls. Studies were excluded if they reported sST2 concentrations only for patients with specific diseases or if they used plasma instead of serum for ST2 measurements. No statistical tests were used for this approach. The results of this experiment are therefore purely descriptive.

## 3. Results

### 3.1. Evaluation (1a): Comparison of Results Using DuoSet ELISA Versus Using Quantikine ELISA

Aliquoted serum samples from the study by Bekos et al. [7] were analyzed using the DuoSet ELISA and the Quantikine ELISA. Detailed results are given in Table 2.

### 3.2. Evaluation (1b): Comparison of DuoSet Standard Versus Quantikine Standard on the Pre-Coated Quantikine Plate

To compare the results of the DuoSet assay with the Quantikine assay, we performed the Quantikine assay according to the manufacturer’s protocol, adding a recombinant standard from the DuoSet. We then calculated sST2 serum concentrations twice: first, using the standard curve calculated for the conventional Quantikine assay, and second, using the standard curve from the DuoSet recombinant standard. The results for both determination methods are presented in Table 3.

Our comparison revealed that all serum samples had higher sST2 concentrations when calculated using the DuoSet recombinant standard than with the corresponding Quantikine recombinant standard. The median fold increase was 4.3.

### 3.3. Evaluation (1b): Comparison of DuoSet Standard Versus Quantikine Standard on DuoSet Plate

To compare the results of the DuoSet assay with the Quantikine assay, we performed the DuoSet assay according to the manufacturer’s protocol and included a recombinant standard from the Quantikine plate. We then calculated sST2 serum concentrations twice: first, using the standard curve calculated for the conventional DuoSet assay, and then using the standard curve from the Quantikine recombinant standard. The results for both determination methods are presented in Table 3. Our analysis showed that, when using a non-precoated plate and following the DuoSet protocol, all sST2 serum concentrations were lower than those obtained on the precoated plate. Additionally, using the corresponding DuoSet recombinant standard, the median values indicated a 1.9-fold increase in serum concentration compared to calculations using the Quantikine recombinant standard on the same plate.

### 3.4. Evaluation (2a): Comparison of sST2 Serum Concentrations Using DuoSet ELISA 2016 vs. Duoset ELISA 2023

To evaluate potential differences in the performance of the DuoSet ELISA between 2016 and 2023, we extracted sST2 serum concentrations from the study by Bekos et al. [7] and re-analyzed corresponding serum aliquots—subjected to no more than two freeze–thaw cycles—using a recently manufactured DuoSet lot. Results obtained with the older DuoSet assay showed a median concentration of 0.09 ng/mL with an interquartile range of 0.07 to 0.1 ng/mL, whereas the recently produced DuoSet assay showed a median concentration of 1.17 ng/mL with an interquartile range of 0.81 to 3.23 ng/mL (Table 4).

### 3.5. Evaluation (2b): Comparison of Previously Published Studies Using DuoSet ELISA and Quantikine ELISA

Over recent decades, published studies in healthy controls show wide variability for both DuoSet and Quantikine assays. We condensed the evidence into a single summary (Table 5) and reported one central-tendency measure and one dispersion measure per study (ng/mL).

## 4. Discussion

In summary, our findings underscore that the results obtained by current ELISAs for measurement of sST2—even those supplied by the same manufacturer—are not comparable. Consistent with our findings, a head-to-head comparison of three sST2 assays (MBL, Presage, and a point-of-care lateral-flow test) reported both constant and proportional bias between methods, indicating that absolute concentrations are not directly comparable [16]. Substantial variability in measured concentrations arises from differences in assay platform (DuoSet development kit—manual coating, biotinylated detection with streptavidin–horseradish peroxidase (streptavidin–HRP)—versus Quantikine pre-coated kit with a horseradish peroxidase (HRP)–conjugated detection antibody), plate preparation/coating method, antibody configuration (pairing, clone and epitope specificity, detection chemistry), calibrator composition (e.g., glycosylated vs. nonglycosylated; full-length vs. fragment; matrix-matched vs. buffer-based), and reagent lot (batch-to-batch variation in antibodies, plates, calibrators, 3,3′,5,5′-tetramethylbenzidine (TMB) substrate, and streptavidin–horseradish peroxidase (streptavidin–HRP)). Moreover, the Duoset ELISA has shown significantly different sST2 concentrations for healthy individuals over the years (Table 5). In addition to the poor comparability of sST2 results obtained using different ELISAs, the concentration shift in a particular assay over time hinders comparisons of sST2 concentrations across different studies. These problems highlight the need for continuous monitoring of assay performance and standardized reference materials. Therefore, we recommend using the same assay lot within a particular study and interpreting cross-study comparisons with caution.

One possible explanation for our findings is the lack of standardized validation procedures for calibrators, which may contribute to variability in sST2 measurements [1,9,17]. Mass spectrometry—particularly isotope-dilution mass spectrometry (ID-MS)—provides highly accurate methods for calibrator validation and reference standard development [18,19,20]. Villanueva (2014) et al. [21] position ID-MS as a reference technique for absolute protein quantification. Yu et al. (2024) [22] demonstrate a validated ID-MS method for IL-6, underscoring the feasibility of SI-traceable measurements, i.e., results traceable to the International System of Units—kg, m, s, mol, etc.—through an unbroken calibration chain with stated uncertainties. In routine clinical chemistry, ID-MS already serves as a reference measurement procedure to assign calibrators and establish reference values (e.g., for steroid hormones and C-peptide) [18,19,20]. In parallel, mass spectrometry can resolve structural attributes—such as site-specific glycosylation or oxidative modifications—that influence antibody-based assay calibration [23].

A recent example of mapping ELISA readouts to absolute concentrations is MASCALE (Mass Spectrometry Enabled Conversion to Absolute Levels of ELISA Antibodies), which combines peptide-specific LC–MS/MS with ELISA to generate conversion curves across platforms and laboratories [24]. Although developed for immunoglobulins, this MS-anchoring framework could be extended to antigen-based assays such as sST2 to support harmonization and mitigate the inter-assay differences observed here [24]. That said, MS-based calibrator validation can be limited by post-translational modifications—especially glycosylation—that impair proteolysis and peptide ionization [23,25,26]. Mitigation strategies include enzymatic deglycosylation, glycopeptide enrichment (e.g., lectin affinity), advanced fragmentation such as Electron Transfer Dissociation (ETD) and Higher-energy Collisional Dissociation (HCD), and, in some workflows, quantification of deglycosylated surrogate peptides [23]. In contrast, amino acid analysis (AAA)—based on complete hydrolysis and quantification of individual amino acids—is independent of protein conformation and matrix effects, and may therefore be more robust for glycoprotein quantification [1,27,28]. Consistent with these considerations, Mueller et al. (2015) [1] noted that even clinically approved sST2 assays such as Presage^®^ lack transparency regarding antibody epitope specificity and calibrator structure, reinforcing the need for SI-traceable reference materials (e.g., via AAA) and harmonized calibration.

An additional source of assay variability lies in the design and epitope specificity of the antibodies used in ELISAs. For commercially available sST2 assays, the exact epitopes are typically undisclosed, and prior reviews have highlighted that differences in antibody binding sites, together with calibrator composition, can drive between-assay divergence [1]. Because sST2 is a glycoprotein, variations in glycan structures or protein folding can alter epitope accessibility and thereby antibody binding. As an analogy, N-glycosylation at a critical site of myelin oligodendrocyte glycoprotein (MOG) was shown to reduce autoantibody binding in a substantial subset of patients, consistent with steric hindrance by glycans [29]. Circulating sST2 can occur in free form or bound to its ligand IL-33; antibodies targeting epitopes within/near the IL-33-binding region may preferentially detect free sST2 and under-recover complexed forms. This form-dependence has been discussed in analytical reviews of sST2 assays and helps explain inter-assay differences [1]. As a clinical illustration, Scott et al. [30] quantified IL-33 forms and the circulating IL-33/sST2 complex in patients, showing that complex-specific readouts can diverge from total sST2. The widely used Presage^®^ assay illustrates these analytical challenges. Reviews note limited public information on the precise epitopes and emphasize that antibody recognition may depend on protein conformation, which, together with calibrator preparation, can influence quantification and comparability. Conceptually, the preferred analytical strategy is to resolve molecular forms—i.e., to quantify free and IL-33-bound sST2 separately using form-discriminating capture/detection reagents or complementary assays. Modern discovery methods—such as phage display and recombinant antibody engineering—enable selection of binders to defined, non-overlapping epitopes and can incorporate positive/negative selection (e.g., panning on free sST2 with counter-panning against the IL-33 complex) to yield antibodies specific for either form [31,32]. For example, Even-Desrumeaux et al. (2014) [33] developed a phage-display strategy applicable to diverse antibody fragments and alternative scaffolds.

Beyond heart failure, sST2 has been implicated across a broad spectrum of inflammatory and immune-mediated diseases, including ARDS [34], sepsis and trauma [6,35], liver failure [8], burn injury [36], COVID-19 [37,38], and autoimmune disorders [12,39,40]. These disease contexts often show dynamic changes in biomarker concentrations, underscoring the need for strict analytical consistency and standardized measurement procedures [41]. In their comprehensive review, the ESC Biomarker Study Group emphasized that many promising biomarkers, including sST2, have not yet fully transitioned into clinical practice—partly due to measurement variability and a lack of standardization—and highlighted the urgent need for harmonized reference standards and validated assay methodologies [4].

Importantly, these assay-related differences are not mere technical curiosities but may carry serious clinical implications. sST2 is increasingly used in heart failure management for therapy monitoring and prognostication [4,35,42,43]. As shown by Januzzi et al. (2015) [42], an sST2 level exceeding 35 ng/mL is associated with increased mortality risk. At the commonly used ~35 ng/mL threshold, Presage and a point-of-care lateral-flow assay classified patients similarly, even though absolute concentrations and analytical sensitivity differed—supporting assay-specific cut-offs rather than cross-platform equivalence [16]. The analytical characteristics of Presage^®^ were previously investigated by Dieplinger et al. (2009) [44], who showed that its monoclonal antibodies recognize structural (non-linear) epitopes whose availability depends on protein folding and purification conditions. This implies that variations in glycosylation, protein conformation, or ligand binding—such as IL-33 interaction—could affect antibody recognition. The lack of publicly available information on epitope specificity and calibrator structure reinforces broader concerns about assay comparability and clinical interpretability. Accordingly, the findings by Dieplinger et al. (2015) [16] further highlight the need for harmonized calibration and well-characterized antibodies to improve cross-platform comparability.

Several other studies have already addressed assay-related variability. For example, Mueller et al. (2012) [9] reported discrepancies of up to 70% in sST2 concentrations between three commercial immunoassays despite using the same serum. These findings mirror our observations and underscore the broader relevance of this issue.

## 5. Limitations

This study has several limitations. First, we did not include patient samples with pathologically elevated sST2, and we lacked earlier (historical) specimens from diseased cohorts. Consequently, we could not determine whether the assay-dependent differences observed here translate into shifts over time or diagnostic and/or prognostic discordance in real-world trajectories (e.g., around clinical cut-offs) in patients. Second, comparisons were restricted to two ELISA systems from a single manufacturer; while this strengthens internal validity, it may limit generalizability to other platforms. Since any degradation would bias concentrations downward, the higher values observed here indicate that the true between-assay difference is larger than estimated. Finally, we did not experimentally distinguish free from IL-33–bound sST2, which could differentially affect antibody recognition and recovery. Despite these constraints, the work is valuable because it (i) documents substantial within-manufacturer variability across assay platform, lot, plate preparation, and calibrator; (ii) reveals plausible concentration shifts over time linked to assay component changes; and (iii) provides practical recommendations for research and clinical laboratories (consistent within-study lots, cautious cross-study comparisons, transparent kit/lot reporting, and consideration of orthogonal anchors for harmonization).

## 6. Conclusions

In conclusion, our study provides strong evidence that currently available ELISA platforms for sST2 measurement—even when produced by the same manufacturer—are not analytically comparable. Substantial variability in measured concentrations arises from differences in assay platform (DuoSet development kit—manual coating, biotinylated detection + streptavidin-HRP—vs. Quantikine pre-coated kit with HRP-conjugated detection antibody), plate preparation/coating method, antibody configuration (pairing, clone/epitope specificity, detection chemistry), calibrator composition (e.g., glycosylated vs. nonglycosylated; full-length vs. fragment; matrix-matched vs. buffer-based), and reagent lot (batch-to-batch variation in antibodies, plates, calibrators, TMB, and streptavidin-HRP). These discrepancies pose significant challenges for cross-study comparability and for the interpretation of sST2 levels in longitudinal settings. Given the increasing clinical use of sST2 for risk stratification and therapy guidance in heart failure [4,35,41,42,43,45] and inflammatory diseases [6,12,39,46,47,48], harmonization efforts are urgently needed. Future approaches should prioritize the development of structurally defined reference materials, the application of orthogonal quantification methods such as mass spectrometry or amino acid analysis, and the design of well-characterized antibodies capable of detecting sST2—free or ligand-bound forms. Without such standardization, even small shifts in assay configuration may lead to misinterpretation of biomarker trends and potentially inappropriate clinical decisions.

## Figures and Tables

**Table 1:** Analytical precision of DuoSet and Quantikine ELISAs (intra-/inter-assay).

ELISA	Precision	Sample	*n*	Mean (ng/mL)	SD (ng/mL)	*CV* (%)
DuoSet	intra-assay	1	2	8.64	0.08	0.93
		2	2	9.24	0.12	1.3
		3	2	8.52	0.12	1.4
	inter-assay	1	2	1.92	0.16	8.2
		2	2	2.44	0.025	1.0
		3	2	0.72	0.11	16.0
Quantikine	intra-assay	1	2	21.8	0.7	3.2
		2	2	22.8	0.41	1.8
		3	2	8.9	0.41	5.6
	inter-assay	1	2	26.4	0.47	1.8
		2	2	39.5	3.1	7.8
		3	2	0.31	0.02	5.3

Values are shown as mean, standard deviation (SD), and coefficient of variation (*CV*%).

**Table 2:** sST2 serum concentrations: DuoSet vs. Quantikine in marathon cohorts.

	Quantikine ELISA		DuoSet ELISA	
Timepoint	Marathoners (*n* = 27)	Half Marathoners (*n* = 34)	Marathoners (*n* = 27)	Half Marathoners (*n* = 34)
pre-run	36.0 [27.9–59.7]	30.3 [21.4–38.8]	20.4 [11.1–26.0]	18.6 [13.7–26.4]
goal	63.6 [36.8–80.9]	35.2 [24.6–54.7]	29.9 [16.0–41.9]	22.6 [15.7–37.4]
post-run	42.9 [29.5–56.0]	26.4 [19.6–41.1]	20.2 [12.6–26.7]	16.5 [12.4–28.4]

Data are given as median (ng/mL) with interquartile range in brackets. The blood samples were drawn immediately before the marathon (pre-run), immediately after crossing the finish line (goal), and 2–7 days after the marathon (post-run).

**Table 3:** Calibrator and plate effects on sST2 concentrations (DuoSet vs. Quantikine).

	*n*	Median (ng/mL)	IQR (ng/mL)	Fold-Change (vs. Reference)
Quantikine plate + Quantikine standard	32	71.5	41.8–115.6	1.0
Quantikine plate + DuoSet standard	32	308.3	106.6–608.6	4.3
DuoSet plate (uncoated) + Quantikine standard	32	5.0	3.7–7.4	1.0
DuoSet plate (uncoated) + DuoSet standard	32	8.0	5.6–11.3	1.9

Fold-change = median (setup) ÷ median (reference) within the same plate. All samples are serum; the identical sample set was measured under different assay/standard combinations; reference: original standard/plate pairing.

**Table 4:** sST2 concentrations (ng/mL) analyzed using R&D DuoSet ELISA from 2016 and 2023.

Duoset ELISA (2016)	DuoSet ELISA (2023)
sST2 Serum Concentrations (ng/mL)	sST2 Serum Concentrations (ng/mL)
0.07	7.11
0.11	2.36
0.08	2.47
0.09	4.03
0.10	3.02
0.08	0.88
0.11	4.34
0.10	4.48
0.06	0.88
0.12	1.17
0.09	0.74
0.10	0.88
0.09	3.44
0.06	0.35
0.09	1.34
0.07	0.97
0.06	0.75
0.06	0.46
0.09	0.70
**0.09 [0.07–0.10]**	**1.17 [0.81–3.23]**

Values in bold format represent the median [IQR] for each group.

**Table 5:** Serum concentrations of sST2 in healthy controls measured with DuoSet ELISA (**a**) and Quantikine ELISA (**b**) in previously published studies.

(**a**)		
**Publications Using DuoSet ELISA**	***n***	**sST2 (ng/mL)**
Brunner et al. (2004) [6]	15	Mean 0.32 SEM ± 0.072
Hacker et al. (2009) [10]	64	Mean 0.09 IQR: 0.012–0.177
Bekos et al. (2016) [7]	30	Mean 0.06 SEM ± 0.008
Urban et al. (2021) [11]	20	Median 18.00 IQR: 12.00–22.00
(**b**)		
**Publications Using Quantikine ELISA**	***n***	**sST2 (ng/mL)**
Han et al. (2017) [12]	27	Mean 7.30 SEM ± 0.22
Yin et al. (2019) [13]	306	Median 549.05 IQR: 427–721
Hannappe et al. (2020) [14]	42	Mean 13.44 SEM ± 7.60
Singh et al. (2024) [15]	57	Mean 25.11 SEM ± 1.76

SEM, standard error of the mean; IQR, interquartile range; ELISA, Enzyme-Linked Immunosorbent Assay; values are given in ng/mL.

## Data Availability

The raw data supporting the conclusions of this article will be made available by the authors on request.

## References

[1] Mueller T., Jaffe A.S. (2015). Soluble ST2—Analytical considerations. Am. J. Cardiol..

[2] Pusceddu I., Dieplinger B., Mueller T. (2019). ST2 and the ST2/IL-33 signalling pathway-biochemistry and pathophysiology in animal models and humans. Clin. Chim. Acta.

[3] Mildner M., Storka A., Lichtenauer M., Mlitz V., Ghannadan M., Hoetzenecker K., Nickl S., Dome B., Tschachler E., Ankersmit H.J. (2010). Primary sources and immunological prerequisites for sST2 secretion in humans. Cardiovasc. Res..

[4] Meijers W.C., Bayes-Genis A., Mebazaa A., Bauersachs J., Cleland J.G.F., Coats A.J.S., Januzzi J.L., Maisel A.S., McDonald K., Mueller T. (2021). Circulating heart failure biomarkers beyond natriuretic peptides: Review from the Biomarker Study Group of the Heart Failure Association (HFA), European Society of Cardiology (ESC). Eur. J. Heart Fail..

[5] Kuroiwa K., Li H., Tago K., Iwahana H., Yanagisawa K., Komatsu N., Oshikawa K., Sugiyama Y., Arai T., Tominaga S.I. (2000). Construction of ELISA system to quantify human ST2 protein in sera of patients. Hybridoma.

[6] Brunner M., Krenn C., Roth G., Moser B., Dworschak M., Jensen-Jarolim E., Spittler A., Sautner T., Bonaros N., Wolner E. (2004). Increased levels of soluble ST2 protein and IgG1 production in patients with sepsis and trauma. Intensive Care Med..

[7] Bekos C., Zimmermann M., Unger L., Janik S., Hacker P., Mitterbauer A., Koller M., Fritz R., Gäbler C., Kessler M. (2016). Non-professional marathon running: RAGE axis and ST2 family changes in relation to open-window effect, inflammation and renal function. Sci. Rep..

[8] Roth G.A., Zimmermann M., Lubsczyk B.A., Pilz J., Faybik P., Hetz H., Hacker S., Mangold A., Bacher A., Krenn C.G. (2010). Up-regulation of interleukin 33 and soluble ST2 serum levels in liver failure. J. Surg. Res..

[9] Mueller T., Zimmermann M., Dieplinger B., Ankersmit H.J., Haltmayer M. (2012). Comparison of plasma concentrations of soluble ST2 measured by three different commercially available assays: The MBL ST2 assay, the Presage ST2 assay, and the R&D ST2 assay. Clin. Chim. Acta.

[10] Hacker S., Lambers C., Pollreisz A., Hoetzenecker K., Lichtenauer M., Mangold A., Niederpold T., Hacker A., Lang G., Dworschak M. (2009). Increased soluble serum markers caspase-cleaved cytokeratin-18, histones, and ST2 indicate apoptotic turnover and chronic immune response in COPD. J. Clin. Lab. Anal..

[11] Urban M.H., Stojkovic S., Demyanets S., Hengstenberg C., Valipour A., Wojta J., Burghuber O.C. (2021). Soluble ST2 and All-Cause Mortality in Patients with Chronic Obstructive Pulmonary Disease-A 10-Year Cohort Study. J. Clin. Med..

[12] Han J.H., Suh C.-H., Jung J.-Y., Ahn M.-H., Kwon J.E., Yim H., Kim H.-A. (2017). Serum Levels of Interleukin 33 and Soluble ST2 Are Associated with the Extent of Disease Activity and Cutaneous Manifestations in Patients with Active Adult-onset Still’s Disease. J. Rheumatol..

[13] Yin X., Cao H., Wei Y., Li H.-H. (2019). Alteration of the IL-33-sST2 pathway in hypertensive patients and a mouse model. Hypertens. Res..

[14] Hannappe M.-A., Arnould L., Méloux A., Mouhat B., Bichat F., Zeller M., Cottin Y., Binquet C., Vergely C., Creuzot-Garcher C. (2020). Vascular density with optical coherence tomography angiography and systemic biomarkers in low and high cardiovascular risk patients. Sci. Rep..

[15] Singh H., Khurana A., Kumar A., Saluja R. (2024). Serum levels of interleukin-33, soluble ST2 and IgE in patients with asthma: A case-control study. J. Asthma.

[16] Dieplinger B., Egger M., Gegenhuber A., Haltmayer M., Mueller T. (2015). Analytical and clinical evaluation of a rapid quantitative lateral flow immunoassay for measurement of soluble ST2 in human plasma. Clin. Chim. Acta.

[17] Aimo A., Januzzi J.L., Bayes-Genis A., Vergaro G., Sciarrone P., Passino C., Emdin M. (2019). Clinical and Prognostic Significance of sST2 in Heart Failure: JACC Review Topic of the Week. J. Am. Coll. Cardiol..

[18] Deng Y., Zhang C., Wang J., Zeng J., Zhang J., Zhang T., Zhao H., Zhou W., Zhang C. (2023). An Accurate Isotope Dilution Liquid Chromatography-Tandem Mass Spectrometry Method for Serum C-Peptide and Its Use in Harmonization in China. Ann. Lab. Med..

[19] Cho S.-E., Han J., You J., Lee J.H., Yi A., Lee S.G., Lee E.H. (2025). Development and Validation of a Novel Isotope Dilution-Ultraperformance Liquid Chromatography-Tandem Mass Spectrometry Method for Serum C-Peptide. Ann. Lab. Med..

[20] Gradl K., Taibon J., Singh N., Albrecht E., Geistanger A., Pongratz S., Hutzler S., Mayer M., Kleinschmidt C., Geletneky C. (2020). An isotope dilution LC-MS/MS-based candidate reference method for the quantification of androstenedione in human serum and plasma. Clin. Mass. Spectrom..

[21] Villanueva J., Carrascal M., Abian J. (2014). Isotope dilution mass spectrometry for absolute quantification in proteomics: Concepts and strategies. J. Proteom..

[22] Yu Z., Wang J., Xia W., Wang Y., Zhang Y., Tang J., Cui H., Yang X., Bao C., Ye Z. (2024). The Development of an Isotope Dilution Mass Spectrometry Method for Interleukin-6 Quantification. Int. J. Mol. Sci..

[23] Liu H., Zhang N., Wan D., Cui M., Liu Z., Liu S. (2014). Mass spectrometry-based analysis of glycoproteins and its clinical applications in cancer biomarker discovery. Clin. Proteom..

[24] Tang C., Verwilligen A., Sadoff J., Brandenburg B., Sneekes-Vriese E., van den Kerkhof T., Dillen L., Rutten L., Juraszek J., Callewaert K. (2024). Absolute quantitation of binding antibodies from clinical samples. npj Vaccines.

[25] Illiano A., Pinto G., Melchiorre C., Carpentieri A., Faraco V., Amoresano A. (2020). Protein glycosylation investigated by mass spectrometry: An overview. Cells.

[26] Lee J.Y., Kim J.Y., Park G.W., Cheon M.H., Kwon K.-H., Ahn Y.H., Moon M.H., Lee H.-J., Paik Y.K., Yoo J.S. (2011). Targeted mass spectrometric approach for biomarker discovery and validation with nonglycosylated tryptic peptides from N-linked glycoproteins in human plasma. Mol. Cell. Proteom..

[27] Kambhampati S., Li J., Evans B.S., Allen D.K. (2019). Accurate and efficient amino acid analysis for protein quantification using hydrophilic interaction chromatography coupled tandem mass spectrometry. Plant Methods.

[28] Reinmuth-Selzle K., Tchipilov T., Backes A.T., Tscheuschner G., Tang K., Ziegler K., Lucas K., Pöschl U., Fröhlich-Nowoisky J., Weller M.G. (2022). Determination of the protein content of complex samples by aromatic amino acid analysis, liquid chromatography-UV absorbance, and colorimetry. Anal. Bioanal. Chem..

[29] Marti Fernandez I., Macrini C., Krumbholz M., Hensbergen P.J., Hipgrave Ederveen A.L., Winklmeier S., Vural A., Kurne A., Jenne D., Kamp F. (2019). The glycosylation site of myelin oligodendrocyte glycoprotein affects autoantibody recognition in a large proportion of patients. Front. Immunol..

[30] Scott I.C., van Zuydam N., Cann J.A., Negri V.A., Tsafou K., Killick H., Liu Z., McCrae C., Rees D.G., England E. (2025). IL-33 is associated with alveolar dysfunction in patients with viral lower respiratory tract disease. Mucosal Immunol..

[31] Zarei B., Javidan Z., Fatemi E., Rahimi Jamnani F., Khatami S., Khalaj V. (2020). Targeting c-Met on gastric cancer cells through a fully human fab antibody isolated from a large naive phage antibody library. Daru.

[32] Ledsgaard L., Ljungars A., Rimbault C., Sørensen C.V., Tulika T., Wade J., Wouters Y., McCafferty J., Laustsen A.H. (2022). Advances in antibody phage display technology. Drug Discov. Today.

[33] Even-Desrumeaux K., Nevoltris D., Lavaut M.N., Alim K., Borg J.-P., Audebert S., Kerfelec B., Baty D., Chames P. (2014). Masked selection: A straightforward and flexible approach for the selection of binders against specific epitopes and differentially expressed proteins by phage display. Mol. Cell. Proteom..

[34] Alladina J.W., Levy S.D., Hibbert K.A., Januzzi J.L., Harris R.S., Matthay M.A., Thompson B.T., Bajwa E.K., National Heart, Lung, and Blood Institute Acute Respiratory Distress Syndrome Network (2016). Plasma Concentrations of Soluble Suppression of Tumorigenicity-2 and Interleukin-6 Are Predictive of Successful Liberation From Mechanical Ventilation in Patients With the Acute Respiratory Distress Syndrome. Crit. Care Med..

[35] Davini F., Fogolari M., D’Avanzo G., Ristori M.V., Nucciarelli S., Bani L., Cristiano A., De Cesaris M., Spoto S., Angeletti S. (2025). Soluble suppression of tumorigenicity 2 (sst2) as a diagnostic and prognostic marker in acute heart failure and sepsis: A comparative analysis. Diagnostics.

[36] Hacker S., Dieplinger B., Werba G., Nickl S., Roth G.A., Krenn C.G., Mueller T., Ankersmit H.J., Haider T. (2018). Increased serum concentrations of soluble ST2 predict mortality after burn injury. Clin. Chem. Lab. Med..

[37] Omland T., Prebensen C., Jonassen C., Svensson M., Berdal J.E., Seljeflot I., Myhre P.L. (2021). Soluble ST2 concentrations associate with in-hospital mortality and need for mechanical ventilation in unselected patients with COVID-19. Open Heart.

[38] Park M., Hur M., Kim H., Lee C.H., Lee J.H., Kim H.W., Nam M., Lee S. (2023). Soluble ST2 as a Useful Biomarker for Predicting Clinical Outcomes in Hospitalized COVID-19 Patients. Diagnostics.

[39] Hong Y.-S., Moon S.-J., Joo Y.-B., Jeon C.-H., Cho M.-L., Ju J.H., Oh H.-J., Heo Y.-J., Park S.-H., Kim H.-Y. (2011). Measurement of interleukin-33 (IL-33) and IL-33 receptors (sST2 and ST2L) in patients with rheumatoid arthritis. J. Korean Med. Sci..

[40] Kim D.-J., Baek S.-Y., Park M.-K., Park K.-S., Lee J.H., Park S.-H., Kim H.-Y., Kwok S.-K. (2013). Serum level of interleukin-33 and soluble ST2 and their association with disease activity in patients with Behcet’s disease. J. Korean Med. Sci..

[41] Meijers W.C., van der Velde A.R., Muller Kobold A.C., Dijck-Brouwer J., Wu A.H., Jaffe A., de Boer R.A. (2017). Variability of biomarkers in patients with chronic heart failure and healthy controls. Eur. J. Heart Fail..

[42] Januzzi J.L., Mebazaa A., Di Somma S. (2015). ST2 and prognosis in acutely decompensated heart failure: The International ST2 Consensus Panel. Am. J. Cardiol..

[43] Piper S., deCourcey J., Sherwood R., Amin-Youssef G., McDonagh T. (2016). Biologic variability of soluble ST2 in patients with stable chronic heart failure and implications for monitoring. Am. J. Cardiol..

[44] Dieplinger B., Januzzi J.L., Steinmair M., Gabriel C., Poelz W., Haltmayer M., Mueller T. (2009). Analytical and clinical evaluation of a novel high-sensitivity assay for measurement of soluble ST2 in human plasma—The Presage ST2 assay. Clin. Chim. Acta.

[45] Traxler D., Zimmermann M., Simader E., Veraar C.M., Moser B., Mueller T., Mildner M., Dannenberg V., Lainscak M., Jug B. (2020). The inflammatory markers sST2, HSP27 and hsCRP as a prognostic biomarker panel in chronic heart failure patients. Clin. Chim. Acta.

[46] Haider T., Simader E., Hacker P., Ankersmit H.J., Heinz T., Hajdu S., Negrin L.L. (2018). Increased serum concentrations of soluble ST2 are associated with pulmonary complications and mortality in polytraumatized patients. Clin. Chem. Lab. Med..

[47] Artru F., Bou Saleh M., Maggiotto F., Lassailly G., Ningarhari M., Demaret J., Ntandja-Wandji L.-C., Pais de Barros J.-P., Labreuche J., Drumez E. (2020). IL-33/ST2 pathway regulates neutrophil migration and predicts outcome in patients with severe alcoholic hepatitis. J. Hepatol..

[48] Ohta N., Yasudo H., Mizutani M., Matsushige T., Fukano R., Kittaka S., Maehara K., Ichihara K., Ohga S., Hasegawa S. (2019). Serum soluble ST2 as a marker of renal scar in pediatric upper urinary tract infection. Cytokine.

